# J'accuse

**DOI:** 10.1100/tsw.2000.15

**Published:** 2000-12-01

## Abstract

The accusations were really flying this week. At global warming negotiations in The Hague, Nature's lead story reports the Europeans accused the U.S. of intransigence in the face of a looming catastrophe and the U.S. shot back that the Europeans are ignoring solid environmental science. Science leads with a group of scientists who are accusing their own community of (occasional) misconduct.

*Nature* kicks off the week with an article about the collapse of the international negotiations to reduce greenhouse gas emissions. (*Science* also covers the story, but in its number 2 slot.) The meeting's stated goal was to reach an agreement on methods of attaining the carbon-emission reductions agreed upon three years ago in Kyoto, Japan, report *Nature* and *Science*. But talks collapsed as sleep-deprived negotiators wilted in the face of an intransigent U.S. The delegation from the States wants to include the effects of carbon dioxide absorbing forests and farmlands in the calculation of a country's total emissions. Both journals reported that a last-minute U.S. concession failed to convince European negotiators, who rejected the proposal. "There was a lack of time and a lack of trust," said Jennifer Morgan of the World Wildlife Fund in Science. Talks will probably resume in May and *Nature* put a positive spin on the story, saying that there is great hope that this meeting has paved the way for a spring compromise.

Sex leads in *Science* this week. Most people are reluctant to talk about their sex lives, so sociologist Charles Turner was surprised when one of his data collectors started reporting too many successful interviews for their National Institute of Health study of human sexual behavior. Turns out, the researcher was lying. *Science* reports that Turner told his story last month at the redundantly titled Office of Research Integrity's (ORI) Research Conference on Research Integrity held in Research ...oops, that's one too many... in Bethesda, MD. The scientists met to ponder the question: How often does misconduct occur? There appears to be no consensus on the answer, reports *Science*. Estimates range from 1 fraud per 100,000 scientists per year to the 1 in 100 researchers who "consistently report" knowing about an instance of misconduct. The problem appears to start early. *Science* says that biologist Elizabeth Davidson and colleagues at Arizona State University in Tempe found that 84 to 91% of introductory biology and zoology students admitted to manipulating lab data "almost always" or "often," usually to get a better grade. Duh.

NASA took a beating in both magazines this week. After the space agency cancelled a planned nanorover mission to an asteroid — the budget had tripled from an estimated $20 million to over $60 million — the American Astronomical Society's (AAS) 1200-member planetary sciences division called for an end to the delays and cancellations, report the two magazines. Just this year, NASA has killed three other planetary science missions. The AAS and NASA agree there is a problem, but disagree on how to fix it. AAS officials called for external review, report both magazines, while NASA wants to clean its own house.
